# Observational study protocol: the faecal microbiome in the acute stage of new-onset paediatric type 1 diabetes in an Irish cohort

**DOI:** 10.1136/bmjopen-2024-089206

**Published:** 2025-01-30

**Authors:** Elaine Catherine Kennedy, Fiona Catherine Ross, Carol-Anne O’Shea, Aonghus Lavelle, Paul Ross, Eugene Dempsey, Catherine Stanton, Colin Patrick Hawkes

**Affiliations:** 1 Department of Paediatrics and Child Health, University College Cork, Cork, Ireland; 2 INFANT Research Centre, University College Cork, Cork, Ireland; 3 Department of Anatomy & Neuroscience, University College Cork, Cork, Ireland; 4 APC Microbiome Ireland, University College Cork, Cork, Ireland; 5 Department of Neonatology, Cork University Maternity Hospital, Cork, Ireland; 6 Teagasc Food Research Centre Moorepark, Moorepark, Ireland; 7 Perelman School of Medicine, University of Pennsylvania, Philadelphia, Pennsylvania, USA

**Keywords:** DIABETES & ENDOCRINOLOGY, MICROBIOLOGY, Paediatric endocrinology

## Abstract

**Abstract:**

**Introduction:**

Type 1 diabetes (T1D) is an autoimmune-mediated disorder caused by the destruction of pancreatic beta cells. Although there is an underlying genetic predisposition to developing T1D, the trigger is multifactorial and likely includes environmental factors. The intestinal microbiome has been identified as one such factor. Previous studies have illustrated differences in the microbiota of people with T1D compared with healthy controls. This study aims to describe the evolution of the microbiome and metabolome during the first year of clinical T1D, or stage 3 T1D diagnosis, and investigate whether there are differences in the microbiome and metabolome of children who present with and without diabetic ketoacidosis. The study will also explore possible associations between the microbiome, metabolome, glycaemic control and beta cell reserve.

**Methods and analysis:**

This prospective cohort study will include children with newly diagnosed T1D and sibling controls (n=100, males and females) and their faecal microbiome will be characterised using shotgun metagenomic sequencing at multiple time points during the first year of diagnosis. We will develop a microbial culture biobank based on culturomic studies of stool samples from the healthy controls that will support future investigation. Metabolomic analysis will aim to identify additional biomarkers which may be involved in disease presentation and progression. Through this initial exploratory study, we aim to identify specific microbial biomarkers which may be used as future interventional targets throughout the various stages of T1D progression.

**Ethics and dissemination:**

This study has been approved by the Clinical Research Ethics Committee of the Cork Teaching Hospitals. Study results will be available to patients with T1D and their families, carers, support networks and microbiome societies and other researchers.

**Trial registration number:**

The clinicaltrials.gov registration number for this trial is NCT06157736.

**STRENGTHS AND LIMITATIONS OF THIS STUDY**

- The inclusion of sibling controls will allow for comparison of the microbiome and metabolome to help identify unique biomarkers at type 1 diabetes (T1D) onset.
- Longitudinal aspect of this study allows monitoring of the evolution of the microbiome and metabolome over the first year of T1D disease.
- This is a single-site study.

## Introduction

 The incidence of type 1 diabetes (T1D) has been increasing over the past few decades, especially in young children.^1^,^6^ T1D is caused by autoimmune-mediated destruction of pancreatic beta cells. This leads to a complete deficiency of insulin with affected individuals requiring lifelong exogenous insulin administration to achieve glucose homeostasis. The underlying aetiology of this condition is poorly understood but includes a genetic predisposition with a subsequent presumed environmental trigger^7 8^ that initiates and perpetuates the T-cell-mediated autoimmune process.^9^

Several large cohort studies have identified differences in the oral and faecal microbiome in those with T1D compared with healthy controls and suggest that microbiome composition may have a role in the pathogenesis of T1D.^10^,^14^ The composition of the intestinal microbiota is intimately linked to intestinal permeability and inflammation, providing a plausible causal association with T1D development. Under specific conditions, disturbance in the regular microbiota colonising the intestinal tract can occur, thus contributing to the onset of numerous diseases such as obesity,^15^ rheumatoid arthritis,^16^ non-alcoholic fatty liver disease,^17^ cardiovascular diseases,^18^ inflammatory bowel disease^19^ and psychiatric disorders.^20^ T1D may be among this list of diseases caused, or modulated by changes in the intestinal microbiota, supported by observational studies of children who develop T1D.^21^ Under such conditions, the proliferation of proinflammatory bacteria may occur through interactions with the intestinal epithelium. This interaction triggers the release of cytokines, activating essential inflammatory pathways, thereby heightening morbidity and extending the duration of critical illness.^22^

### The intestinal microbiome through the stages to T1D development

The development of T1D progresses through stages including genetic risk, immune activation, immune response, diabetes-related autoantibody development and the progressive decline in insulin production.^23^ This progression from genetic risk to established T1D can be characterised by four stages.^24^ Stage 1 is the presence of multiple islet autoantibodies. Stage 2 includes the presence of abnormal glucose tolerance. Stage 3 involves hyperglycaemia, and stage 4 is defined as established T1D. Associated microbiome characteristics have been described as early as the immune activation stage. A small study of 10 children with one positive diabetes-related autoantibody (ie, the immune activation stage) demonstrated increased intestinal permeability and distinct differences in their intestinal microbiota compared with healthy controls.^25^ A low abundance of lactate- and butyrate-producing species has also been demonstrated.^26^ These microbiome features also disrupt intestinal epithelial barrier function. The converse appears to be true also, where intestinal microbiomes containing genes that promote the biosynthesis of short-chain fatty acids (SCFAs) and strengthen the intestinal barrier appear to have a protective effect against developing autoimmunity or T1D.^12^

At the time of diagnosis, small case-control studies have demonstrated a reduced Firmicutes/Bacteroides ratio,^27^,^29^ reduced microbial diversity^30^ and higher proportion of non-buyrate-producing lactate-using bacteria^13^ in children with T1D. Lower numbers of specific species of *Clostridium* (clusters IV and XIVa),^10 31^ *Prevotella*^13^ and *Akkermansia*^13^ have also been identified in children with new-onset T1D. Rates of intestinal *Candida* colonisation are also higher in those with new-onset^32^ or established^33^ T1D. Additionally, the oral microbiome has been studied in patients with T1D with significant differences demonstrated between those with T1D and healthy controls.^14^

The intestinal microbiome may represent an important environmental trigger for T1D development. At the genus level, *Bacteroides* are frequently found to be present in higher abundance in the intestine of people with T1D or beta cell autoimmunity.^13 26 29 34 35^ *Bacteroides* species might contribute to the thinning of the intestinal mucus layer, increasing intestinal permeability and inflammation.^35^ Increased intestinal permeability can facilitate bacterial and dietary antigens to translocate into the circulation, promoting systemic inflammation.^21^ However, Cinek *et al* showed that *Bacteroides caccae* was inversely correlated with the onset of beta cell autoimmunity.^36^ Different mechanisms have been proposed to explain the role of *Bacteroides* in T1D. Vatanen *et al* hypothesised that the production of a lipopolysaccharide with immunoinhibitory properties by *Bacteroides dorei* strains in early life alters immune development, contributing to the development of T1D.^37^ The conversion of lactate to butyrate by intestinal bacteria increases mucin synthesis. However, *Bacteroides* species convert lactate to acetate, propionate and succinate instead, reducing mucin synthesis and contributing to the thinning of the intestinal mucus layer.^13^

The progression of T1D is affected by the levels of circulating glutamic acid decarboxylase (GAD) antibodies.^38^ GAD is commonly produced by pancreatic beta cells. It has been suggested that GABA production through the bacterial GAD system can lead to the miseducation of the host immune system, triggering autoimmunity against pancreatic beta cells, via molecular mimicry.^21^ Knowing that GABA production by *Bacteroides* GAD-system is a common trait in the human intestine,^39^ these findings suggest that *Bacteroides* may play a role in T1D progression. *Phocaeicola dorei* and *Phocaeicola vulgatus* were classified as *Bacteroides* until recently,^40^ so most studies investigating *Bacteroides* associations with T1D also include *Phocaeicola* species. Different studies found that *P. vulgatus* and *P. dorei* species were in lower relative abundance in patients with T1D than in control groups.^31 34 41^ However, Davis-Richardson *et al* found that *P. dorei* and *P. vulgatus* dominate the intestinal microbiota of Finnish children before the development of islet autoimmunity, making the role of *Phocaeicola* spp. in the disease unclear.^42^

### Intestinal microbiome and permeability in T1D

The ‘leaky gut’ hypothesis links the microbiome with the pathogenesis of T1D.^43^,^45^ The intestinal microbial ecosystem plays an active role in both immune responses and intestinal barrier integrity.^46^ Increased intestinal permeability is seen in rat models at high risk for developing T1D,^47 48^ and increases in gastric and small intestinal permeability are seen prior to the development of insulinitis and diabetes.^47^ In transgenic mice carrying a T cell receptor for a beta cell autoantigen, antibiotic-induced depletion of the intestinal microbiota can trigger the development of T1D,^49^ suggesting a protective role of the intestinal microbiota. In humans, increased intestinal permeability has been demonstrated through increased mannitol^50^ or lactulose^51^ absorption, or serum zonulin levels^52^ at the time of diagnosis of T1D. Furthermore, children at risk for developing T1D have increased intestinal permeability and distinct differences in their intestinal microbiomes compared with healthy controls.^25^ Gastroenteritis caused by rotavirus can trigger seroconversion in children at risk for developing T1D,^53^ and this mechanism may include microbiome disruption.

### Metabolomic associations with the intestinal microbiome

Although characteristic features of the microbiome have been described in small studies comparing children with T1D and controls, additional information is required to understand the role of the microbiome in this context. Metabolic profiles of taxa present in the microbiome may help investigate microbial functional significance^54^ and possibly lead to the identification of therapeutic targets within microbial metabolic pathways.^55^ There have been limited studies exploring metabolomic profiles in children with T1D, but triglycerides, methionine and phospholipids have been shown to possibly play a role in the seroconversion stage of T1D development.^56^ Following the development of T1D-related autoantibodies in infants, analysis of stool metabolomic profiles in conjunction with microbiome analysis demonstrates a drop in alpha diversity with inflammation-favouring metabolites.^57^ This further supports the hypothesis that the intestinal microbiome represents a potential target in altering the progression of T1D.

In order to elucidate associations between the microbiome and the occurrence and evolution of T1D, establish mechanistic links and identify possible targets for interventions, we plan to initially perform short-read metagenomic sequencing followed by bioinformatic analysis. Additionally, we will establish untargeted metabolomic profiles followed by validation of biomarkers of interest. This correlation with microbiome profiles will establish potential microbial functions linking our observations with associated metabolic activities. Specifically, characteristics of the microbiome at the onset of T1D could potentially be linked to immune^9 58 59^ and microRNA^60^,^62^ markers. The interplay between these markers and the microbiome will further advance our understanding of potential mechanisms underlying any associations identified in this study.

By describing and analysing unique patterns of faecal microbiome biodiversity at the onset of T1D, this study will provide a basis for further interventional studies that aim to halt or modify the trajectory of disease progression. This appears to be a feasible target of future study as there are small but promising studies that have identified the intestinal microbiome as a potential target for intervention in T1D.

Prior studies^25^,^33^ employed microbiome analysis techniques such as real-time quantitative PCR, 16S sequencing and culturing of stool samples to gain insights into microbial changes associated with T1D. A recent study has suggested correlation between the microbiome and T1D progression, and reported an association between C peptide levels and microbial diversity.^63^ The novelty of the present study lies in its longitudinal and in-depth approach, employing short-read shotgun metagenomic sequencing in a large cohort during the early stages of T1D. Enabling the examination of entire genomes, this methodology allows for the comprehensive analysis of both the composition and functionality of the faecal microbiota from the onset of disease diagnosis and throughout the first year. Additionally, culturing of stool samples, in parallel with metagenomic shotgun sequencing, will enable insights into the potential identification and characterisation of essential missing microbes during the first year of T1D progression for potential therapeutic interventions.

Advancing our understanding of the intestinal microbiome in this way may have therapeutic implications. In children with established T1D, prebiotics were associated with increased endogenous insulin production and a possible improvement in intestinal permeability.^64^ Restoring the intestinal microbiome via faecal transplant in adults with new-onset T1D has been shown to delay the decline in beta cell function,^65^ suggesting that modulating the intestinal microbiome in those with newly diagnosed T1D may impact disease progression. Identifying putative therapeutic targets within the microbiome would allow for a more focused approach that could be effective in this population. While these studies provide compelling data in support of an association between the microbiome and T1D, further data are required to identify specific microbiome interventional targets throughout the stages of diabetes progression.

This study is designed to bridge existing knowledge gaps and incorporates a robust methodology with specific strengths, augmenting the significance of its findings. The proposed study’s specimen collection strategy, implemented at various time points over a 1-year period, aims to deepen our comprehension of the temporal dynamics of colonising microbiota and their interactions with host physiology. While multicentre trials capturing sufficient case numbers of T1D cases will offer robust conclusions, this study will offer valuable information on faecal microbiome and metabolome associations with disease progression in new-onset T1D. Subsequent research studies can leverage these findings to explore treatment strategies, potentially offering recommendations for the effective administration of probiotic strains and supplements to enhance faecal microbiota diversity and function for improved outcomes.

### Objectives and outcomes

The primary aim of this study is to describe the evolution of the microbiome and metabolome in children during their first year with stage 3 T1D, compared with their siblings without diabetes.

Secondary objectives and outcomes:

1. To establish if there are differences in the microbiome or metabolome of children who present in diabetic ketoacidosis (DKA) versus those who present without DKA.
2. To establish if there are differences in the microbiome or metabolome in those presenting with mild, moderate or severe DKA.
3. To establish if there is an association between the microbiome or metabolome and glycaemic control during the first year of diagnosis.
4. To establish if there is an association between the microbiome or metabolome and beta cell reserve 1 year after T1D diagnosis.
5. To establish if there is an association between the microbiome or metabolome at the time of T1D diagnosis and preceding hyperglycaemia.

## Methods and analysis

This study will recruit 50 children at the time of diagnosis and 50 unaffected sibling controls and will employ a prospective cohort study methodology to investigate the faecal microbiome of children with newly diagnosed T1D in the first year of diagnosis. All children with newly diagnosed T1D will be assessed for eligibility for enrolment in this study. Inclusion and exclusion criteria are outlined separately in the recruitment strategy outline. Parents of eligible cases and controls will be approached for consent. If a case does not have siblings, they will still be eligible for recruitment. If a case has multiple siblings, more than one sibling can be recruited as a control. Recruitment will be conducted over a 1-year period. Consent for enrolment for both cases and controls will be obtained during the first hospital visit on diagnosis of the index case. The study started in October 2023 and is planned to finish by September 2026.

At each time point (0, 4, 8, 52 weeks), stool will be collected for microbiome analysis from all cases and controls (n=100). Urine for metabolomics will be collected from all study participants at the same time points. A food frequency questionnaire will be completed by all cases and controls at the first and fourth study visits (0 and 52 weeks, respectively). Clinical data, including insulin dosing, glycaemic control and blood results, will be documented at each visit for cases. Cases will be asked to participate in a mixed meal tolerance test (MMTT) at study visit four (52 weeks). This will quantify stimulated C peptide. This result will help to establish if there is a correlation between microbiome and metabolome evolution and beta cell reserve at 1 year. Shotgun metagenome sequencing will be conducted on faecal samples and urine metabolomic analysis will be employed to detect metabolic biomarkers. The study will explore the impact of the underlying diagnosis and environmental factors. The mode of delivery plays a crucial role in shaping the composition and function of the neonatal microbiome.^66 67^ Caesarean section delivery affects early microbiota transmission from mother to infant and the subsequent priming of the immune system, with delivery-dependent differences early in gut microbiota colonisation continuing through the first year of life.^66 67^ Due to this, we will record the mode of delivery in our data collection with ethical approval to access patient charts and will perform shotgun sequencing to compare cases with age-matched controls from 6 months onwards, should any become available.

In addition, the establishment of a microbial biobank will be achieved based on culturomics of stool samples from healthy sibling controls solely as a source of microbes to develop a microbial culture biobank that will support future investigations. Culturomics is solely performed on faecal samples from healthy siblings, and not index cases, with the hope to culture and preserve beneficial bacterial strains that are present in the healthy microbiome but potentially missing or depleted in individuals with T1D. We hypothesise that the microbiome of sibling controls, which is likely healthier than that of the index cases, may contain key bacterial species involved in immune regulation and metabolic health that are absent or depleted in individuals with T1D. By focusing on healthy controls, we aim to identify beneficial microbial species that may play a protective role in delaying or preventing the onset of T1D. The preservation and study of these strains hold the potential for developing therapeutic targets aimed at slowing the onset and progression of the disease.

### Public and patient involvement

During study development, parents were invited to discuss the study and provide feedback through an online virtual meeting. This input was integrated into the study design prior to finalising the approach.

### Study participation and recruitment of subjects

This is a prospective observational cohort study including children with newly diagnosed T1D and their healthy sibling controls. Participants will be recruited from the diabetes service at Cork University Hospital (CUH). We aim to recruit approximately 50 of these children (cases). Healthy siblings of cases will be eligible to be recruited as controls for the study, and we aim to recruit approximately 50 controls. The inclusion and exclusion criteria are summarised in table 1. The study timeline is shown in table 2.

**Table 1:** Inclusion and exclusion criteria

Cases	Controls
*Inclusioncriteria*
Males and females with newly diagnosed T1D	First-degree family members of cases
Aged between 6 months and 18 years at their first study visit	Do not have T1D
Consent provided by parent/guardian to participate in this study	Aged at least 6 months and no older than 18 years at the first study visit
Age-dependent assent or consent obtained for participants aged 6–18 years	Consent provided by parent/guardian to participate in this study
*Exclusioncriteria*	
Are less than 6 months or older than 18 years at the time of their first study visit	
Individuals who have a known diagnosis of inflammatory bowel disease or another medical condition which the study team deems might interfere with the microbiome	
Individuals with complex medical or behavioural needs that would deem the participant unable to participate in the study	
Have a significant acute or chronic coexisting illness (cardiovascular, gastrointestinal, endocrinological, immunological, metabolic) or any condition which contraindicates entry to the study according to the investigator’s judgement	
Participants who are receiving treatment involving experimental medications	
Have a malignant disease or any concomitant end-stage organ disease	

T1Dtype 1 diabetes

**Table 2:** Study visits

*Cases*				
Visit number	1	2	3	4
Study week	1	4	8	52
Informed consent/assent	X			
Review inclusion/exclusion criteria	X			
Demographics/medical history	X			
Height and weight	X	X	X	X
Clinical data extraction (insulin doses, glycaemic control, blood results)	X	X	X	X
Food frequency questionnaire	X			X
Laboratory tests				
Stool for microbiome analysis	X	X	X	X
Urine for metabolomic analysis	X	X	X	X
Mixed Meal Test for C peptide				X
Serum for HLA-typing				X
*Controls*				
Visit number	1	2	3	4
Study week	1	4	8	52
Informed consent/assent	X			
Review inclusion/exclusion criteria	X			
Demographics/medical history	X			
Height and weight				X
Food frequency questionnaire	X			X
Laboratory tests				
Stool for microbiome analysis	X	X	X	X
Urine for metabolomic analysis	X	X	X	X
Serum for HbA1c measurement				X
Serum for HLA-typing				X
Serum for diabetes autoantibodies				X

### Screening and visit 1

Screening will take place during the case’s admission to hospital following their diagnosis with T1D. After reviewing the inclusion and exclusion criteria, once a participant is deemed eligible, they are then offered participation in the study. The aim of the study and the procedures to be undertaken will be explained to all potential participants and their parents/guardians, and they will be provided with a Patient Information Leaflet (PIL) and an Informed Consent Form (ICF) and an age-appropriate Information Leaflet and Assent Form for the participant if relevant. The researcher will review the information outlined in the PIL and the age-appropriate information leaflet with the parent/guardian and potential participant and answer any questions they might have. If the child and their parents/guardians agree to take part in the study, they will be asked to read and sign the assent form and the ICF, respectively. Participants will receive a signed copy to keep. Demographics and medical history, height and weight measurements will be taken at this visit. A food frequency questionnaire will be completed by cases and controls during this visit. Samples of stool and urine will also be collected from cases and controls at this visit. The date of sampling is recorded. Patient status, including whether or not they were acidotic at the time of sampling, will be noted. Clinical data, including insulin dosing and glycaemic control and blood results of cases, will be documented at this visit. Parents or guardians will also be interviewed regarding their child’s history of medication use. The information pertaining to any medications administered will be documented in the participant’s medical records, when accessible, and in the study’s Case Report Form (CRF).

### Study visits 2 and 3

The second and third study visits will take place when the participant (case) returns to CUH for their routine follow-up appointment with the Diabetes Team approximately 1 and 2 months after T1D diagnosis. At these visits, cases and controls will be asked to provide stool and urine samples to the study team. Clinical data, including insulin dosing and glycaemic control, will be documented. Cases will be examined and height and weight recorded.

### Study visit 4

The fourth study visit will take place approximately 12 months after T1D diagnosis. At this visit, cases and controls will be asked to provide stool and urine samples to the study team. They will also be asked to complete a food frequency questionnaire. Clinical data, including insulin dosing, glycaemic control and blood results, will be documented. Height and weight will be recorded for all study participants. Cases will undergo their MMTT at this visit. If the case opts out of the 2-hour MMTT, they can have a shortened test where a single blood draw is taken at 90 min. If the case does not wish to take part in any MMTT, the results of routine clinical bloods, including fasting glucose and fasting C peptide levels, will be recorded instead. This will help to establish whether there is a correlation between microbiome and metabolome evolution and beta cell reserve. Serum for HLA-typing will be taken at this visit also.

Controls will undergo phlebotomy for HLA-typing, diabetes autoantibodies and HbA1c analysis at this study visit. Parent(s) will be given these results if requested. There will be flexibility in the timing of phlebotomy for controls enrolled in the study. Their blood can be taken at any stage during the study (between study visit 1 and the end of the study) to ensure it is convenient for the family and the control.

### Subject withdrawal/exclusion

In accordance with the Declaration of Helsinki, the participant can discontinue their involvement in the study at any point if either they or their parent/guardian wishes to be withdrawn from the study.

Participants may be excluded from the study if they exhibit a condition that contradicts the initial criteria or if, at any stage, they are deemed unfit to continue the study at the investigator’s discretion. Any decision to withdraw will be documented in the source records and the relevant CRF. Subsequently, individuals who choose to withdraw from the study will be substituted. Samples from those who opt to no longer participate in the study will be preserved and may undergo analysis within the research. Furthermore, participants who are excluded from the study after giving their consent will also be replaced.

### Sample collection and analysis

#### Sample collection

##### Stool and urine

Stool and urine samples will be collected by the participant (or parent/guardian depending on the age of the participant) in the hospital using a collection kit and instructions that will be provided. If the samples are required to be collected at home, participants are informed that the samples provided should be as fresh as possible, ideally within 12 hours or less. Index cases and sibling control urine samples are collected in standard Steriline containers. Index cases are instructed to place their stool samples into a standard collection box, while the stool samples of sibling controls are collected anaerobically using the GutAlive anaerobic stool collection kit for culturomics. The GutAlive kit preserves the viability of anaerobic bacteria without altering the overall microbiome composition, ensuring that valid comparisons can be made between index cases and sibling controls despite the slight difference in sample collection methods. This distinction in collection methods was necessary due to the differing objectives of the study for each group; however, both groups follow consistent handling and storage protocols. The stool samples are then placed in an empty biohazard bag. It is then stored at 4°C. Freezer bags are provided to all participants to ensure samples remain cold when being transported to the hospital. A research nurse transfers samples from the participants’ homes to the laboratory if the participants are not scheduled for a hospital appointment close to the time of sample collection.

Once at the laboratory, the stool and urine samples are stored at −80°C for long-term preservation. Stool samples from sibling controls, collected anaerobically using the GutAlive anaerobic stool collection kit, aim to maintain microbial integrity and allow for the culturing of highly sensitive anaerobic bacteria. Anaerobic samples are immediately cultured under anaerobic conditions on arrival to the laboratory, while the remaining stool is stored at −80°C for future analysis. Anaerobic stool samples are serially diluted and plated on five different media (Brain Heart Infusion; De Man, Rogosa and Sharpe; Tryptic Soy Broth; Yeast Casitone Fatty Acids; M17 Medium; Gifu Anaerobic Medium) to target a broad spectrum of taxa.

##### Serum for diabetes autoantibodies

Serum will be collected from controls at study visit 4 to look for the presence of diabetes autoantibodies. This test will have been carried out in all cases, as per standard medical practice when first diagnosed with T1D.

##### Serum for HbA1C

A blood sample will be collected from controls to measure their HbA1C at study visit 4.

##### Serum for HLA-typing

Approximately 5 mL of serum will be collected from cases and controls at study visit 4.

##### Mixed meal tolerance test

Cases will be asked to participate in a MMTT at study visit 4 at CUH. They will be asked to fast from 22:00 the night before the test but continue to take their normal long-acting insulin, including on the morning of the test. Cases on an insulin pump can continue to take their normal background (basal) insulin before, during and after the test.

To proceed with the test, the case’s glucose level must be between 4 and 11.1 mM before the beginning of the test. Serum for HLA-testing will be taken from the intravenous line during the test.

### Sample analysis

Stool will be analysed by the APC Microbiome Ireland laboratory. Stool samples will be stored at −80°C. The extraction of DNA from faecal matter will be conducted using the QIAmp Fast DNA Stool Mini Kit (QIAGEN, Manchester, UK), which includes an initial bead-beating step in the process.^68^ Microbiome analysis of stool will be performed using shotgun metagenomics sequencing. Initially, the DNA will undergo fragmentation, where it is sheared into fragments of approximately 400–600 bp and tagged with Illumina adaptor sequences. Subsequently, an indexing PCR will be carried out to uniquely index each sample, facilitating the pooling of samples on a single-flow cell and subsequent bioinformatic demultiplexing. Following Illumina Nextera XT protocols, PCR amplicons will be cleaned and normalised. To ensure an even distribution of samples for pooling, qPCR will be conducted. All metagenomic samples will be sequenced on the Illumina NextSeq platform. Raw reads will be filtered based on quality, quantity and length of reads, as well as the removal of human reads. Stool microbiota composition and functionality will be analysed using state-of-the-art methods. Faecal SCFAs will be measured in all study participants as SCFAs have previously been reported to have a protective effect against developing autoimmunity.^69^,^72^ Faecal SCFAs will be analysed by gas chromatography flame ionisation detection using a Varian 3800 GC system, fitted with a DB-FFAP column (30 m L × 0.32 mm ID × 0.25 µm df; Agilent, California, USA) and a flame ionisation detector. Urine for metabolomics will be stored at −80°C and later analysed for untargeted metabolomics at University College Cork. Mass spectrometry methods for biomarker identification, followed by targeted/hypothesis-driven metabolomics methods for biomarker verification, will be conducted.

Statistical instruments for the analysis of microbiome data sets are continually being updated, and we will use the latest methodology available. Part of the faecal samples will be grown for analyses of culturable bacterial strains.

Serum for diabetes autoantibodies will be analysed by Exeter Clinical Laboratory in the UK. Serum for HLA-typing will be analysed at the National Blood Centre, James’ Street, Dublin 8. Serum for HbA1C measurement, glucose and C peptide analysis will be analysed at CUH. Samples will be stored securely in a UCC monitored biobank until transfer for analytics to the relevant laboratory.

### Bioinformatics and statistical analysis

#### Demographic and clinical data

Demographic and clinical data, as well as laboratory information, will be subjected to a normality test using the Shapiro-Wilk test. Descriptive statistics will be employed to characterise data that conform to a normal distribution and will be presented as mean ± SD. Continuous data lacking a normal distribution will be presented using the median and IQRs. Categorical variables will be conveyed as counts and percentages. Group comparisons will involve χ^2^ tests for categorical variables and independent-sample Student’s t-tests for continuous variables meeting the normal distribution criteria. In the case of variables that do not adhere to a normal distribution, the Mann-Whitney U test will be employed.

#### Microbiome analysis

Metagenomic shotgun sequencing data will be analysed using bioBakery suite of tools (https://huttenhower.sph.harvard.edu/biobakery_workflows/). Trimmed and human reads filtered using KneadData (Harvard School of Public Health, Boston, Massachusetts, USA) with the default parameters. Quality-controlled data will be taxonomically profiled at the species level with relative abundance by MetaPhlAn4. Functional profiling will be performed using HUMAnN3 (Harvard School of Public Health, Boston, Massachusetts, USA)^73^ and strain profiling using StrainPhlAn (University of Trento, Trento, Italy).^74^

#### Data collection and management

During the study, all data entered in the CRF will be pseudonymised with a unique identification code allocated at the time of enrolment into the study, and stored in a REDCap database. Identifiable data will be stored separately to this information. Only members of the research team will have access to these identifiers.

The electronic Investigator Site File will be maintained and updated as required during the study. Computers used to collate the data will have limited access measures by encryption codes, username and passwords. Access will be restricted to authorised study personnel only. All identifiable data will be kept for 10 years as per our university Code of Research Conduct. Clinical information will not be released without the written permission of the participant, except as necessary for monitoring, auditing or inspection by the relevant authorities. Published results will not contain any personal data that could allow identification of individual participants. All investigators and associated staff have completed good clinical practice in research training and will comply strictly with the requirements of the General Data Protection Regulation regarding the collection, storage, processing and disclosure of personal information and will uphold the Act’s core principles.

## Ethics and dissemination

This study has been approved by the Clinical Research Ethics Committee of the Cork Teaching Hospitals. The outcomes of the study will be accessible to individuals diagnosed with T1D, along with their families, caregivers, support networks, microbiome societies and other researchers. The findings from the study aim to enhance our comprehension of the faecal microbiota in childhood T1D. Additionally, they will contribute valuable insights for managing the disease, including strategies to prioritise the preservation of faecal microbiota integrity.

### Status of the study

The trial is ethically approved (registration number NCT06157736), and recruitment has commenced.
