# Supplementary material for: Observational study protocol: the faecal microbiome in the acute stage of new-onset paediatric type 1 diabetes in an Irish cohort
Source: BMJ Open. 2025 Jan 30;15(1):e089206. doi: 10.1136/bmjopen-2024-089206 (PMC11784173; doi:10.1136/bmjopen-2024-089206)
Supplement: Supplementary file 1 [file bmjopen-2024-089206.reviewer_comments.pdf]

PEER REVIEW HISTORY

BMJ Open publishes all reviews undertaken for accepted manuscripts. Reviewers are asked to complete a checklist review form and are provided with free text boxes to elaborate on their assessment. These free text comments are reproduced below.

ARTICLE DETAILS

Title (Provisional)

Observational study protocol: The faecal microbiome in the acute stage of new onset paediatric type 1 diabetes in an Irish cohort

Authors

Kennedy, EC; Ross, FC; O'Shea, Carol-Anne; Lavelle, A; Ross, Paul; Dempsey, E; Stanton , Catherine; Hawkes, CP

VERSION 1 - REVIEW

**Reviewer**	**1**
**Name**	**de Beaufort, Carine**
**Affiliation**	**Clinique pédiatrique de Luxembourg, DCCP**
**Date**	**10-Aug-2024**
**COI**	**no**

The paper 'An observational study of the faecal microbiome in the acute stage of new onset paediatric type 1 diabetes' by Kennedy EC, et al describes the protocol to evaluate and compare the role of the microbiome in newly onset stage 3 T1D children and their siblings without known diabetes

The topic is interesting and relevant. But more details are needed to evaluate the robustness of the data and potential analysis.

Abstract

introduction .

With the new classification of T1DM the wording 'early stages' is confusing as the apparently the authors discuss Stage 3 and not the stage 1-2 diabetes. This should be reworded. (Insel RA, et al Staging presymptomatic type 1 diabetes: a scientific statement of JDRF, the Endocrine Society, and the American Diabetes Association. Diabetes Care. 2015 Oct;38(10):1964-74. doi: 10.2337/dc15-1419. PMID: 26404926; PMCID: PMC5321245

Introduction

The objective is to investigate associations between the occurrence of T1D and the microbiome. Based on the current knowledge, the authors should include the internationally accepted staging (1-3) of type 1 diabetes, and should discuss whether the microbiome during study period relates to (past) hyperglycemia and potentially not to the early stages (stage1-2) with only immune perturbations.

Methodology

The choice of the family as control is endorsed as here the difference based on the disease may be best identified (Heintz-Buschart A et al. Nat Microbiol. 2016 doi: 10.1038/nmicrobiol.2016.180.). The controls should be checked for auto antibodies as in this age group risk for Stage 1 and 2 T1D is present. This is only planned at the end of the follow up. The authors should discuss what will be done with the data collected on those who may be positive for one or more antibodies.

In the medical history it is advised to include the mode of delivery (C-section or vaginally, Wampach, L et al . Birth mode is associated with earliest strain-conferred gut microbiome functions and immunostimulatory potential. Nat Commun 9, 5091 (2018). https://doi.org/10.1038/s41467-018-07631-:) as this has an impact on the evolving microbiome .

The microbiome over the first year(s) of life changes, and cases and controls can be included from 6 months onwards : how will this be addressed? (Busi, et al. Persistence of birth mode-dependent effects on gut microbiome composition, immune system stimulation and antimicrobial resistance during the first year of life. ISME COMMUN. 1, 8 (2021) https://doi.org/10.1038/s43705-021-00003-5)

It should be discussed up to how many days after the diagnosis , and whether during or after the acute DKA period, samples are collected or whether normalisation of the metabolic status is obtained at the T0 collection.

With respect to the choice of the collection of samples, it seems surprising that the remission period is not included as this is mostly seen at a later time period. (Podolakova, K.,. et al. Complete remission in children and adolescents with type 1 diabetes mellitus—prevalence and factors. (Sci Rep 13, 6790 (2023). https://doi.org/10.1038/s41598-023-34037-7)

FFQ will be the full one? Or the 24h recall? Is this age specific?

Pre analytical conditions of the stool collection/urine collection need to be detailed.

With respect to the sampling the pre analytic conditions are critical and determine whether the functional microbiome can be analysed or whether only shotgun analysis can be done. This is not specified in sufficient details and it is unclear whether the samples are collected snap frozen or not, or whether a sampling tube with stabilizer is provided (which will allow the shotgun metagenomic analysis, but it will not give the full picture of the functional microbiome . If the samples are collected at home it should be clear for how much time they

may be exposed to the air or room temperature. If the stool sample is cultured , it should be clarified how the researchers will address the analysis of those taxa which can not be cultured or would request very specific conditions.

Discussion

The discussion should include the recently published outcome of the INNODIA study , which provides some insights in the first 24 months after diagnosis and the relationship with glycemic control (Vatanen, T et al. Gut microbiome shifts in people with type 1 diabetes are associated with glycaemic control: an INNODIA study. Diabetologia (2024). https://doi.org/10.1007/s00125-024-06192-7)

The comparison with family members is a strength of this study.

Finally it is important to discuss to what extend the microbiome in stage 3 of T1DM can provide insights in the pathogenesis of the condition or whether it investigates the interaction of the microbiome and hyperglycemia.

**Reviewer**	**2**
**Name**	**Honkanen, Jarno**
**Affiliation**	**University of Helsinki**
**Date**	**22-Aug-2024**
**COI**	**I declare no competing interests.**

The current study will provide valuable information about the changes of the gut microbial composition during the first year after clinical diagnosis of type 1 diabetes. The study potentially reveals new microbial factors that may play a physiological role in regulating the clinical outcome of a newly diagnosed patient.

Overall the study is well designed and executed. However, a couple of details should be clarified prior publishing the study.

Firstly, it is not fully clear why 'culturomics' is only performed from the fecal samples of healthy siblings. Rationale for this should be opened in the manuscript.

Secondly, the manuscript should contain a brief description how the data protection and management is taken into consideration. A technical description of a safe data storage and management environment and other procedures, in accordance to General Data Protection Regulation (GDPR), should be opened in the manuscript.

VERSION 1 - AUTHOR RESPONSE

Reviewer: 1
Dr. Carine de Beaufort, Clinique pédiatrique de Luxembourg
Comments to the Author:
The paper 'An observational study of the faecal microbiome in the acute stage of new onset paediatric type 1 diabetes' by Kennedy EC, et al describes the protocol to evaluate and compare the role of the microbiome in newly onset stage 3 T1D children and their siblings without known diabetes
The topic is interesting and relevant. But more details are needed to evaluate the robustness of the data and potential analysis.

Abstract
introduction .
With the new classification of T1DM the wording 'early stages' is confusing as the apparently the authors discuss Stage 3 and not the stage 1-2 diabetes. This should be reworded. (Insel RA, et al Staging presymptomatic type 1 diabetes: a scientific statement of JDRF, the Endocrine Society, and the American Diabetes Association. Diabetes Care. 2015 Oct;38(10):1964-74. doi: 10.2337/dc15-1419. PMID: 26404926; PMCID: PMC5321245

Thank you for this comment. We agree the words "early stages" may lead to some confusion. We have re-worded this as suggested.
"This study aims to describe the evolution of the microbiome and metabolome during the first year of T1D diagnosis and to investigate if there are differences in the microbiome and metabolome of children who present with and without diabetic ketoacidosis (DKA)."

Introduction
The objective is to investigate associations between the occurrence of T1D and the microbiome. Based on the current knowledge, the authors should include the internationally accepted staging (1-3) of type 1 diabetes, and should discuss whether the microbiome during study period relates to (past) hyperglycemia and potentially not to the early stages (stage1-2) with only immune perturbations.

Thank you for this comment. The primary objective of our study is to describe the evolution of the microbiome (and metabolome) during the first year of T1D diagnosis (stage 3), and to compare this evolution to the microbiome of healthy sibling controls.

In light of your suggestion, we have included the internationally accepted staging of T1D in our manuscript.

Whilst not in our initial objectives, we will be able to compare the microbiome with HbA1C at diagnosis as a proxy for prior hyperglycaemia, as you have suggested.

The intestinal microbiome through the stages to T1D development

The development of T1D progresses through stages including genetic risk, immune activation, immune response, diabetes-related autoantibody development, and the

progressive decline in insulin production[23]. This progression from genetic risk to established T1D can be characterised by four stages[24]. Stage 1 is the presence of multiple islet autoantibodies. Stage 2 includes the presence of abnormal glucose tolerance. Stage 3 involves hyperglycemia, and stage 4 is defined as established T1D.

*" **Objectives and Outcomes***
The primary aim of this study is to describe the evolution of the microbiome and metabolome during stage 3 of T1D, compared to sibling controls.

Secondary objectives and outcomes:

1. *To establish if there are differences in the microbiome or metabolome of children who present in diabetic ketoacidosis (DKA) versus those who present without DKA.*
2. *To establish if there are differences in the microbiome or metabolome in those presenting with mild, moderate, or severe DKA.*
3. *To establish if there is an association between the microbiome or metabolome and glycaemic control during the first year of diagnosis.*
4. *To establish if there is an association between the microbiome or metabolome and beta cell reserve one year after T1D diagnosis*
5. *To establish if there is an association between the microbiome or metabolome at the time of T1D diagnosis and preceding hyperglycaemia"*

Methodology
The choice of the family as control is endorsed as here the difference based on the disease may be best identified (Heintz-Buschart A et al. Nat Microbiol. 2016 doi: 10.1038/nmicrobiol.2016.180.). The controls should be checked for auto antibodies as in this age group risk for Stage 1 and 2 T1D is present. This is only planned at the end of the follow up. The authors should discuss what will be done with the data collected on those who may be positive for one or more antibodies.

Thank you for this comment. We have included our plan to disclose the results of HbA1c levels, autoantibody status and HLA-typing to parents who request them on page 12 of the manuscript. Although it would have been preferable to measure these levels early in the study, we wanted to maximise the chance of recruiting sibling controls by offering the families flexibility in the timing of these investigations. The controls are healthy children who do not attend the hospital frequently, so we have offered the families involved some flexibility to improve participation.

Controls will undergo phlebotomy for HLA-typing, Diabetes Autoantibodies and HbA1c analysis at this study visit. Parent(s) will be given these results if requested. There will be flexibility in the timing of phlebotomy for controls enrolled in the study. Their blood can be taken at any stage during the study (between Study Visit 1 and the end of the study), to ensure it is convenient for the family and the control.

In the medical history it is advised to include the mode of delivery (C-section or vaginally, Wampach, L et al . Birth mode is associated with earliest strain-conferred gut microbiome functions and immunostimulatory potential. Nat Commun 9, 5091 (2018). https://doi.org/10.1038/s41467-018-07631-:) as this has an impact on the evolving microbiome . The microbiome over the first year(s) of life changes, and cases and controls can be included from 6 months onwards : how will this be addressed? (Busi, et al. Persistence of birth mode-dependent effects on gut microbiome composition, immune system stimulation and antimicrobial resistance during the first year of life. ISME COMMUN. 1, 8 (2021) https://doi.org/10.1038/s43705-021-00003-5)

We are recording the mode of delivery in our data collection. We have ethical approval to access patient charts in order to record this information. We acknowledge that the microbiome over the first year(s) of life changes, and will employ shotgun sequencing to compare cases with age-matched controls from 6 months onwards, should any become available.

"The mode of delivery plays a crucial role in shaping the composition and function of the neonatal microbiome[66,67]. Caesarean section delivery affects early microbiota transmission from mother to infant and the subsequent priming of the immune system, with delivery-dependent differences in early gut microbiota colonization continuing through the first year of life. Due to this, we will record the mode of delivery in our data collection with ethical approval to access patient charts and will perform shotgun sequencing to compare cases with age-matched controls from 6 months onwards, should any become available."

It should be discussed up to how many days after the diagnosis , and whether during or after the acute DKA period, samples are collected or whether normalisation of the metabolic status is obtained at the T0 collection.

Thank you for raising this important point.

Once enrolled in the study, participants are requested to collect a sample from the first stool they pass (T0). Urine is collected at approximately the same time. We understand that not all children will pass stool every day, and there may be practical reasons why the first stool may not be collected by the family. We still wanted to include these subjects in our study. Therefore, we have not limited study participation to those who provide a stool sample within a certain period of time after diagnosis.

In our analysis, we will note the timing of the T0 sample in relation to the date of T1D diagnosis. We will also correlate this analysis with the metabolic status of the case at the time of sampling. Specifically, we will correlate microbiome results with whether or not acidosis had corrected at the time of sampling. We have amended our manuscript (page 11 under the heading 'screening and Visit 1') to reflect this:

“Samples of stool and urine will also be collected from cases and controls at this visit. The date of sampling is recorded. Patient status including whether or not they were acidotic at the time of sampling will be noted.”

With respect to the choice of the collection of samples, it seems surprising that the remission period is not included as this is mostly seen at a later time period. (Podolakova, K.,. et al. Complete remission in children and adolescents with type 1 diabetes mellitus—prevalence and factors. (Sci Rep 13, 6790 (2023). https://doi.org/10.1038/s41598-023-34037-7)

Thank you for this comment. By following study participants for a year from the date of diagnosis, we hypothesise that some cases should be in remission. At the time of recruitment, participants are also asked to consent to future contact for further research. This will allow us to collect more samples later in the disease process if we find that our study period misses the remission phase of disease.

From the ethically-approved consent forms:
I agree to be re-contacted by researchers about possible future research related to the current study for which I may be eligible ***YES/NO***

FFQ will be the full one? Or the 24h recall? Is this age specific?

The Food Frequency Questionnaire used in our study is not age specific. It is a full questionnaire.

Pre analytical conditions of the stool collection/urine collection need to be detailed. With respect to the sampling the pre analytic conditions are critical and determine whether the functional microbiome can be analysed or whether only shotgun analysis can be done. This is not specified in sufficient details and it is unclear whether the samples are collected snap frozen or not, or whether a sampling tube with stabilizer is provided (which will allow the shotgun metagenomic analysis, but it will not give the full picture of the functional microbiome . If the samples are collected at home it should be clear for how much time they may be exposed to the air or room temperature. If the stool sample is cultured , it should be clarified how the researchers will address the analysis of those taxa which can not be cultured or would request very specific conditions.

We have now included details of the pre-analytical conditions on page 15 of the manuscript, under the heading ‘Sample collection and analysis’.

Sample collection and analysis.

Sample Collection
Stool and Urine: Stool and urine samples will be collected by the participant (or parent/guardian depending on the age of the participant) in the hospital using a collection

kit and instructions will be provided. If the samples are required to be collected at home, participants are informed that samples provided should be as fresh as possible, ideally within 12 hours or less. Participants are instructed to place the stool samples into a red bag and put it in a cool place such as the fridge. Freezer bags are provided to all participants to ensure samples remain cold when being transported to the hospital. A research nurse transfers samples from the participants' homes to the laboratory if the participants are not scheduled for a hospital appointment close to the time of sample collection.

Once at the laboratory, the stool and urine samples are stored at -80°C for long-term preservation. Stool samples from healthy controls are collected anaerobically using the GutAlive anaerobic stool collection kit to maintain microbial integrity and allow for the culturing of highly sensitive, anaerobic bacteria. Anaerobic samples are immediately cultured under anaerobic conditions upon arrival to the laboratory, while the remaining stool is stored at -80°C for future analysis. Anaerobic stool samples are serially diluted and plated on five different media (BHI: Brain Heart Infusion, MRS: De Man, Rogosa, and Sharpe, TSB: Tryptic Soy Broth, YCFA: Yeast Casitone Fatty Acids, M17 Medium, GAM: Gifu Anaerobic Medium) to target a broad spectrum of taxa.

Discussion
The discussion should include the recently published outcome of the INNODIA study , which provides some insights in the first 24 months after diagnosis and the relationship with glycemic control (Vatanen, T et al. Gut microbiome shifts in people with type 1 diabetes are associated with glycaemic control: an INNODIA study. Diabetologia (2024). https://doi.org/10.1007/s00125-024-06192-7)

Thank you for highlighting this recently published, relevant study. We have amended our manuscript to include this paper (page 8 of our manuscript).

Prior studies[25-33] employed microbiome analysis techniques such as real-time quantitative polymerase chain reaction, 16S sequencing, and culturing of stool samples to gain insights into microbial changes associated with T1D. A recent study has suggested correlation between the microbiome and T1D progression, and reported an association between c-peptide levels and microbial diversity[63].

The comparison with family members is a strength of this study.
Finally it is important to discuss to what extend the microbiome in stage 3 of T1DM can provide insights in the pathogenesis of the condition or whether it investigates the interaction of the microbiome and hyperglycemia.
Thank you for raising this interesting point.

We agree that it would be difficult to prove that the microbiome in stage 3 of T1DM can be linked with the pathogenesis of disease, as there are multiple genetic and environmental factors involved.

By characterising the microbiome at the time of T1DM diagnosis, we hope to establish if there is an association between the microbiome at that timepoint and preceding hyperglycaemia, as measured by HbA1C. By describing the evolution of the microbiome over the first year of disease, we hope to establish if there is an association between the microbiome and (1) glycaemic control during year one of disease, and (2) beta cell reserve one year after T1DM diagnosis, as quantified with a mixed meal tolerance test.

Objectives and Outcomes

The primary aim of this study is to describe the evolution of the microbiome and metabolome during stage 3 of T1D, compared with sibling controls.

Secondary objectives and outcomes:

1. *To establish if there are differences in the microbiome or metabolome of children who present in diabetic ketoacidosis (DKA) versus those who present without DKA.*
2. *To establish if there are differences in the microbiome or metabolome in those presenting with mild, moderate, or severe DKA.*
3. *To establish if there is an association between the microbiome or metabolome and glycaemic control during the first year of diagnosis.*
4. *To establish if there is an association between the microbiome or metabolome and beta cell reserve one year after T1D diagnosis*
5. *To establish if there is an association between the microbiome or metabolome at the time of T1D diagnosis and preceding hyperglycaemia*

Reviewer: 2
Dr. Jarno Honkanen, University of Helsinki
Comments to the Author:
The current study will provide valuable information about the changes of the gut microbial composition during the first year after clinical diagnosis of type 1 diabetes. The study potentially reveals new microbial factors that may play a physiological role in regulating the clinical outcome of a newly diagnosed patient.
Overall the study is well designed and executed. However, a couple of details should be clarified prior publishing the study.
Firstly, it is not fully clear why 'culturomics' is only performed from the fecal samples of healthy siblings. Rationale for this should be opened in the manuscript.

Thank you for this comment. We have amended our manuscript to include details as to why culturomics is only performed on faecal samples from healthy controls (page 10 of our manuscript, under the heading "Methods and Analysis').

"Culturomics is solely performed on faecal samples from healthy siblings with the hopes to culture and preserve bacterial strains that are missing or depleted in individuals with T1D, that are present in healthy controls. By focusing on healthy controls, we aim to identify beneficial microbial species that may play a protective role in delaying or preventing the onset of T1D. The preservation and study of these strains hold the potential for developing therapeutic targets aimed at slowing the onset and progression of the disease."

Secondly, the manuscript should contain a brief description how the data protection and management is taken into consideration. A technical description of a safe data storage and management environment and other procedures, in accordance to General Data Protection Regulation (GDPR), should be opened in the manuscript.

Thank you for this comment. We have now included a paragraph on our data collection and management.

Data Collection and Management

During the study, all data entered in the case report form (CRF) will be pseudonymised with a unique identification code allocated at the time of enrolment into the study, and stored in a REDCap database. Identifiable data will be stored separately to this information. Only members of the research team will have access to these identifiers.

The electronic Investigator Site File (eISF) will be maintained and updated as required during the study. Computers used to collate the data will have limited access measures by encryption codes, username and passwords. Access will be restricted to authorised study personnel only. All identifiable data will be kept for 10 years as per our university Code of Research Conduct. Clinical information will not be released without the written permission of the participant, except as necessary for monitoring, auditing, or inspection by the relevant authorities. Published results will not contain any personal data that could allow identification of individual participants. All investigators and associated staff have completed good clinical practice in research training, and will comply strictly with the requirements of the General Data Protection Regulation (GDPR) regarding the collection, storage, processing, and disclosure of personal information and will uphold the Act's core principles.

VERSION 2 - REVIEW

**Reviewer**	**1**
**Name**	**de Beaufort, Carine**

**Affiliation**	**Clinique pédiatrique de Luxembourg, DCCP**
**Date**	**13-Nov-2024**
**COI**	

The authors have addressed most of the points raised.

• Re the abstract : I suggest to add 'first year of clinical T1D , or stage 3 T1D diagnosis'

• Re objectives and outcomes i suggest to add 'evolution of.... in children during their first year with stage 3 T1D , compared to their siblings without diabetes.

• Re pre analytical conditions. Please clarify and include in the text whether samples of index cases and siblings are obtained in different y the same way. This is not clear from the current description. (only siblings would use the GutAlive set ?)

• Not using a snap freezing technique will limit the diversity of the composition of the microbiome as the anaerobic population will change

• Using different techniques/tubes for the 2 sets of samples will render comparison impossible.

• Please specify whether the 'red bags' will contain a stabiliser to allow the analysis of metagenomics, (even if meta omics will not be possible).

• It is not clear why different procedures are applied for sibs compared to index cases and it seems important to discuss this better.

VERSION 2 - AUTHOR RESPONSE

Reviewer: 1
Dr. Carine de Beaufort, Clinique pédiatrique de Luxembourg
Comments to the Author:
The authors have addressed most of the points raised.

• Re the abstract : I suggest to add 'first year of clinical T1D , or stage 3 T1D diagnosis'

Thank you for this suggestion. We have amended the abstract as requested.

"This study aims to describe the evolution of the microbiome and metabolome during the first year of clinical T1D, or stage 3 T1D diagnosis, and to investigate if there are differences in the microbiome and metabolome of children who present with and without diabetic ketoacidosis (DKA)."

• Re objectives and outcomes i suggest to add ‘evolution of.... in children during their first year with stage 3 T1D , compared to their siblings without diabetes.

As suggested, we have amended the ‘Objectives and Outcomes’:

“Objectives and Outcomes

The primary aim of this study is to describe the evolution of the microbiome and metabolome in children during their first year with stage 3 T1D, compared to their siblings without diabetes.”

• Re pre analytical conditions. Please clarify and include in the text whether samples of index cases and siblings are obtained in different y the same way. This is not clear from the current description. (only siblings would use the GutAlive set ?)

Thank you for your comment. We appreciate the query regarding the pre-analytical conditions for index cases and healthy siblings. To clarify, while faecal samples from both groups are collected with similar protocols, the samples from sibling controls are collected using the GutAlive kit. This kit does not alter the microbiome composition but maintains an anaerobic environment to preserve live bacteria for culturomics.

For index cases, the use of the GutAlive kit was not necessary, as these samples are not subjected to anaerobic culturomics but rather used for metagenomics. Importantly, the differences in sampling techniques between the groups are specific to the distinct downstream analyses and objectives, and they do not introduce bias in the microbiome composition analysis. This distinction is now clarified in the manuscript.

• Not using a snap freezing technique will limit the diversity of the composition of the microbiome as the anaerobic population will change

Thank you for your comment. We acknowledge that snap freezing is a well-established method for preserving bacterial populations by halting microbial activity. However, in this study, the GutAlive kit was chosen as it is specifically designed to preserve live anaerobic bacteria for culturomics. Using snap freezing for index case samples and the GutAlive kit for control samples would introduce inconsistencies in pre-analytical conditions, potentially limiting direct comparisons between the groups.

• Using different techniques/tubes for the 2 sets of samples will render comparison impossible.

We acknowledge the reviewer’s concern regarding the use of different techniques for the two sets of samples and its potential impact on comparisons. While it is true that healthy sibling samples were collected using the GutAlive kit to preserve an anaerobic environment for culturomics, index case samples were not subjected to culturomics and were instead collected without the GutAlive kit.

However, it is important to note that the use of the GutAlive kit does not alter the microbiome composition itself but merely maintains anaerobic conditions for live bacterial preservation. Consequently, this difference in sampling techniques does not affect the microbiome profile assessed through metagenomic sequencing.

Therefore, comparisons of microbiome composition between index cases and healthy siblings remain valid, as identical protocols will be applied for DNA extraction and shotgun sequencing.

The revised paragraph is below:

"Index cases are instructed to place their stool samples into a standard collection box, while the stool samples of sibling controls are collected anaerobically using the GutAlive anaerobic stool collection kit for culturomics. The GutAlive kit preserves the viability of anaerobic bacteria without altering the overall microbiome composition, ensuring that valid comparisons can be made between index cases and sibling control despite the slight difference in sample collection methods. This distinction in collection methods was necessary due to the differing objectives of the study for each group, however both groups follow consistent handling and storage protocols."

• Please specify whether the 'red bags' will contain a stabiliser to allow the analysis of metagenomics, (even if meta omics will not be possible).

Thank you for this comment. We have amended the text to avoid confusion surrounding the collection boxes. Please note that there is no stabiliser added to the biohazard bags.

"This distinction in collection methods was necessary due to the differing objectives of the study for each group, however both groups follow consistent handling and storage protocols. The stool samples are then placed in an empty biohazard bag. It is then stored at 4 °C."

• It is not clear why different procedures are applied for sibs compared to index cases and it seems important to discuss this better.

Thank you for your comment. The amended paragraph is below:

"Culturomics is solely performed on faecal samples from healthy siblings, and not index cases, with the hopes to culture and preserve beneficial bacterial strains that are present in the healthy microbiome but potentially missing or depleted in individuals with T1D. We hypothesize that the microbiome of sibling controls, which is likely healthier than that of the index cases, may contain key bacterial species involved in immune regulation and metabolic health that are absent or depleted in individuals with T1D. By focusing on healthy controls, we aim to identify beneficial microbial species that may play a protective role in delaying or preventing the onset of T1D. The preservation and study of

these strains hold the potential for developing therapeutic targets aimed at slowing the onset and progression of the disease."

Reviewer: 1
If you have selected 'Yes' above, please provide details of any competing interests.: n/A
